# Bacterial Toxicity of Potassium Tellurite: Unveiling an Ancient Enigma

**DOI:** 10.1371/journal.pone.0000211

**Published:** 2007-02-14

**Authors:** José M. Pérez, Iván L. Calderón, Felipe A. Arenas, Derie E. Fuentes, Gonzalo A. Pradenas, Eugenia L. Fuentes, Juan M. Sandoval, Miguel E. Castro, Alex O. Elías, Claudio C. Vásquez

**Affiliations:** Laboratorio de Microbiología Molecular, Facultad de Química y Biología, Universidad de Santiago de Chile, Santiago, Chile; Tufts University, United States of America

## Abstract

Biochemical, genetic, enzymatic and molecular approaches were used to demonstrate, for the first time, that tellurite (TeO_3_^2−^) toxicity in *E. coli* involves superoxide formation. This radical is derived, at least in part, from enzymatic TeO_3_^2−^ reduction. This conclusion is supported by the following observations made in K_2_TeO_3_-treated *E. coli* BW25113: i) induction of the *ibpA* gene encoding for the small heat shock protein IbpA, which has been associated with resistance to superoxide, ii) increase of cytoplasmic reactive oxygen species (ROS) as determined with ROS-specific probe 2′7′-dichlorodihydrofluorescein diacetate (H_2_DCFDA), iii) increase of carbonyl content in cellular proteins, iv) increase in the generation of thiobarbituric acid-reactive substances (TBARs), v) inactivation of oxidative stress-sensitive [Fe-S] enzymes such as aconitase, vi) increase of superoxide dismutase (SOD) activity, vii) increase of *sodA, sodB* and *soxS* mRNA transcription, and viii) generation of superoxide radical during *in vitro* enzymatic reduction of potassium tellurite.

## Introduction

To date, it is not known if elements like Ag, As, Cd, Cr, Hg, Pb, Te, or some of their derivatives play a defined biological function and they are mainly associated with toxicity [1], [2].

Oxyanions of tellurium, like tellurite (TeO_3_^−2^), are highly toxic for most microorganisms [3]. However, tellurite-resistant bacteria do exist in nature and they often reduce tellurite to its elemental less toxic form Te° that is accumulated as black deposits inside the cell [4], [5].

It has been argued that tellurite toxicity results from its ability to act as a strong oxidizing agent over a variety of cell components [6], [7]. Evidence has accumulated in the last few years suggesting that tellurite could exert its toxicity through intracellular generation of reactive oxygen species (ROS).

ROS compounds such as hydrogen peroxide (H_2_O_2_), superoxide anion (O_2_^−^) and hydroxyl radical (OḢ) are typical byproducts of the aerobic metabolism that can be formed by exposure of cells to free radical-generating molecules like metals and metalloids [8].

Recent indirect evidence suggests a relationship between tellurite toxicity and superoxide generation inside the cell. Tantaleán et al. [9] showed that resistance of *E. coli* to K_2_TeO_3_ increases approximately ten-fold when cells are grown under anaerobic conditions, which is presumably due to the cell's inability to produce ROS under oxygen deprivation conditions. The authors also observed that *E. coli* cells lacking superoxide dismutase genes *sodA* and *sodB* exhibited a tellurite hypersensitive phenotype.

Rojas and Vásquez [10] working with *E. coli* wild type and desulfurase mutants found that most of tellurite toxicity takes place in an aerobic environment. Borssetti et al. [11] reported that *Rhodobacter capsulatus* cells incubated with potassium tellurite exhibit increased superoxide dismutase (SOD) activity and increased resistance to tellurite when exposed to the O_2_^−^ generator paraquat [11].

Here we demonstrate that the oxidative damage attributed to potassium tellurite is due at least in part to the intracellular generation of the reactive oxygen species superoxide radical. We report that K_2_TeO_3_ activates the *ibpA* gene promoter. The *ibpA* gene codes for a small chaperone involved in a heat shock response that has been directly related with O_2_^−^ resistance [12], [13]. In addition, exposure to tellurite generated intracellular reactive oxygen species and increased the cellular content of protein carbonyl groups and thiobarbituric responsive substances (TBARs). Activity of the ROS-sensitive enzyme aconitase decreased upon tellurite exposure, while the activity of superoxide dismutase significantly increased. Moreover, we detected increased levels of *soxS* mRNA after treatment with tellurite. Finally, *soxS, sodA sodB* and *ibpA* mutant strains exhibited a hypersensitive tellurite phenotype when compared to their wild type counterparts.

## Results

### Tellurite triggers expression of the *ibpA* stress response promoter

Aiming to investigate potassium tellurite toxicity in *E. coli* we studied induction of *lacZ* fusions to the well-characterized stress response promoters *ibpA, sulA*, p3*RpoH* and *cspA* in cells exposed to tellurite.

**Figure 1:**
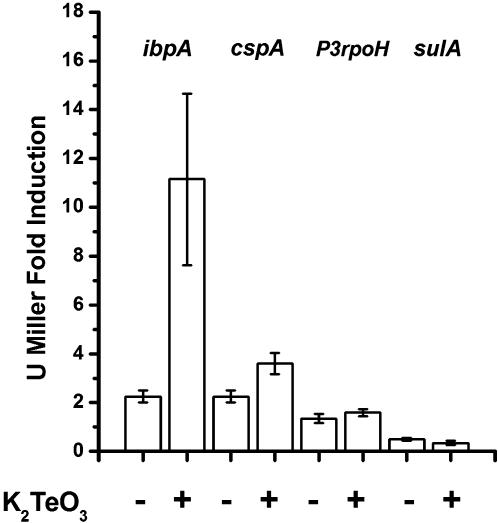
Tellurite-induction of β-galactosidase activity in *E. coli* reporter strains. *E. coli* reporter strains ADA100 [AB734 λΦ(*ibp*::*lacZ*)], ADA310 [AB734λΦ (*cspA*::*lacZ*)], ADA410 [AB734 λΦ(p3*RpoH*::*lacZ*)] and ADA510 [AB734 λΦ(*sulA*::*lacZ*)] containing the stress-responsive promoters *ibpA, cspA, p3RpoH* and *sulA* fused to the *lacZ* gene respectively, were used to study transcription induction in cells treated or untreated with K_2_TeO_3_ (0.5 µg/ml). β-galactosidase activity was evaluated at time 0 and after 3 h with or without tellurite treatment. The fold induction was calculated dividing the value obtained at 3 h by the value at time 0. Results are the average of at least 4 determinations.

Significant promoter activation by K_2_TeO_3_ was observed only for the *ibpA* promoter. Fig. 1 shows that *E. coli* cells treated with tellurite exhibited a 10-fold transcription induction as compared with the untreated controls. This finding is interesting because IbpA protein has been associated with increased resistance to oxidative stress induced by superoxide [12], [13]. CspA mRNA is highly transcribed in response to cytoplasmic protein stress [14], [15]. Our results showed a 2-fold increase in the *cspA* promoter transcription in response to tellurite. Promoters *sulA* and p3*RpoH* did not show any detectable activation suggesting that tellurite does not involve DNA damage or periplasmic stress in *E. coli*. The slight increments of β-galactosidase activity seen in controls after 3 h of tellurite treatment may be due to the fact that the cells have reached the stationary growth phase.

### Tellurite generates ROS in the cytoplasm of *E. coli*

**Figure 2:**
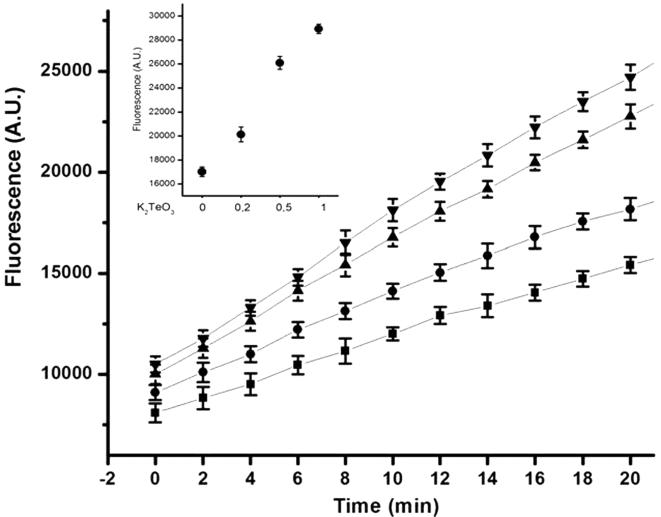
Generation of intracellular ROS by K_2_TeO_3_. Cytoplasmic ROS content was evaluated measuring the H_2_DCFDA probe activation in *E. coli* cells treated with different sub lethal concentrations of tellurite [0 (▪), 0.2 (•), 0.5 (▴) and 1 µg/ml (◂)]. Fluorescence was measured 10 times at 2 min intervals. The inset shows probe activation at 28 min by tellurite at the indicated concentrations. See Methods for details.

The fluorescent probe H_2_DCFDA (2′, 7′- dichlorodihydrofluorescein diacetate) was used to monitor formation of intracellular ROS in tellurite-treated cells (Fig. 2). Cells treated with different sub lethal concentrations of K_2_TeO_3_ exhibited significant probe activation. This activation was proportional to tellurite concentration, with the highest ROS levels in cells treated with 0.5–1.0 µg/ml K_2_TeO_3_. The inset clearly shows that the behaviour of *E. coli* BW25113 remains the same after 28 min of tellurite exposure. The slight increase in probe activation seen in control experiments is most likely related with the generation of metabolic ROS.

These results support previous findings that the oxidative effects of tellurite inside the cell are mediated, at least in part, by an increase in intracellular ROS concentration which in turn is most likely a consequence of tellurite reduction in the bacterial cytoplasm [7], [16].

### Tellurite increases oxidation of cytoplasmatic proteins

**Figure 3:**
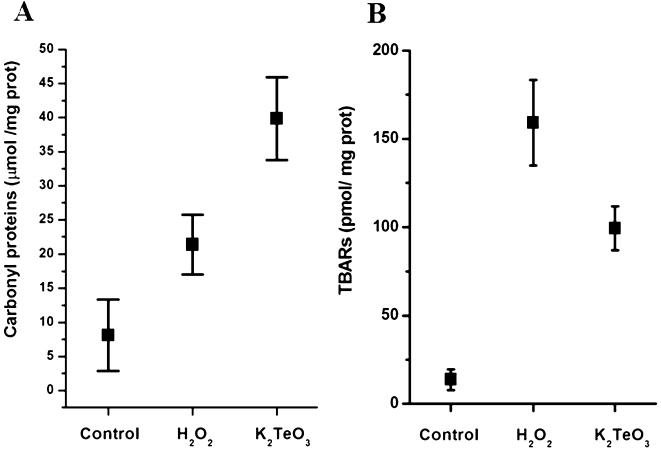
Tellurite increases the oxidation of cytoplasmic proteins and membrane lipids in *E. coli.* Effects of K_2_TeO_3_ (0.5 µg/ml) and H_2_O_2_ (100 µM) on protein carbonyl (A) and TBARs content (B) of *E. coli* BW25113 cells exposed to these compounds for 30 min. A, protein oxidation was determined by a chemical protein carbonyl assay by derivatizing total cellular proteins with DNPH and reading specific carbonyls absorbance at 370 nm. B, membrane peroxidation products were determined as thiobarbituric acid-reactive substances present in crude extracts of *E. coli* BW25113 by the method described by Rice-Evans et al. [19].

The generation of carbonyl groups in proteins, produced by the modification of side chains of some amino acids, is a suitable marker to monitor oxidation of the intracellular environment [17], [18]. Spectrophotometric determination of derivatized carbonyl groups with 2,4 dinitrophenyl hydrazine (DNPH) showed that crude extracts of K_2_TeO_3_-treated *E. coli* exhibited a 4-fold increase in the content of oxidized cytoplasmatic proteins compared with the 2-fold increase observed with peroxide (Fig. 3A). Determination of carbonyl groups using specific antibodies against DNPH-derivatized proteins confirmed the oxidative effect of K_2_TeO_3_ on cytoplasmatic proteins. Although the protein immunoreactive pattern between untreated and treated cells was not significantly different, a few high molecular weight proteins were distinctly observed in tellurite-treated cell extracts (data not shown). Experiments to determine the identity of these proteins are in progress.

### Tellurite increases cytoplasmic TBARs

The level of TBARs has been extensively used to assess the damage of oxidative stress to membrane lipids in many organisms [18], [19]. It has been recently shown that TBARs concentration significantly increases in *E. coli* exposed to hydrogen peroxide [18]. We also observed an increase of TBARs in H_2_O_2_-treated *E. coli* BW25113 (Fig. 3B). However, this increase was only approximately 30% of that previously observed in *E. coli* KS400 [18]. K_2_TeO_3_ treatment also produced an important increase of TBARs in *E. coli* BW25113. This increase was about one half of that induced by H_2_O_2_, an observation that is consistent with the idea of a secondary toxic effect of tellurite due to superoxide anion generation during tellurite reduction.

### Determination of ROS-sensitive and ROS-responsive enzymatic activities

A number of enzymatic activities that are known to be affected by ROS were determined in cells grown under aerobic conditions with or without K_2_TeO_3_ (Table 1). Aconitase activity, a ROS-sensitive enzyme containing a [Fe-S] cluster [17], [20], decreased approximately 5-fold in K_2_TeO_3_ treated cells. Malate dehydrogenase, an enzyme reported to be resistant to oxidative stress [17], [21], showed no differences between tellurite-treated and untreated cells.

**Table 1:** Effect of potassium tellurite on *E. coli* malate dehydrogenase and aconitase*.*

STRAIN	MIC (µg/ml)
wt	2.0
*ibpA*	0.06
*katG*	1.5
*sodAsodB*	0.01
*soxS*	0.75

Enzymatic activities were determined in crude extracts obtained from cells treated or untreated with 0.5 µg/ml K_2_TeO_3_ for 30 min as described in Methods.

**Figure 4:**
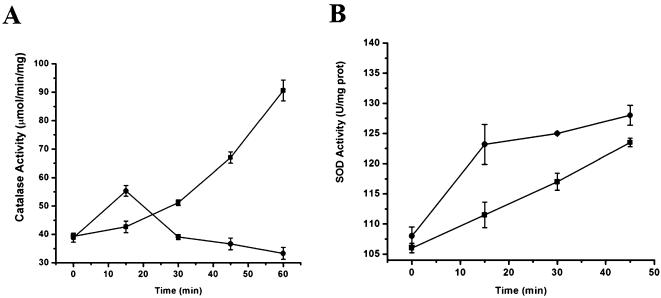
Effect of potassium tellurite on *E. coli* catalase and superoxide dismutase. A, activity of catalases in crude protein extracts of *E. coli* BW25113 treated (•) or untreated (▪) with 0.5 µg/ml of K_2_TeO_3_. Cells were collected at 15 min intervals and catalase activity (µmol hydrogen peroxide/min/mg protein) was determined. B, activity of superoxide dismutases in crude protein extracts of *E. coli* BW25113 treated (•) and untreated (▪) with 0.5 µg/ml K_2_TeO_3_. Cells were harvested and SOD activity (U/mg protein) was determined.

To determine the effect of tellurite on ROS-responsive enzymatic behavior, the activities of catalase (CAT) and superoxide dismutase (SOD) were assayed during the first hour of tellurite treatment. These activities were determined in *E. coli* BW25113 extracts obtained at 15 min intervals after tellurite exposure (Fig. 4).

While catalase activity increased progressively in controls without tellurite, extracts prepared from tellurite-treated *E. coli* cells exhibited a peak of catalase activity during the first 15 min of treatment followed by a 50% decrease in activity (Fig. 4A). This initial rise in activity could be associated with the generation of peroxide produced along the course of superoxide dismutation by the bacterial superoxide dismutase (SOD).

Crude extracts obtained from tellurite-treated cells showed an important increase in SOD activity as compared to the basal level exhibited by untreated cells (Fig. 4B). The highest rise in SOD activity was observed within the first 15 min and was followed by a lower activity increase. These results show that O_2_^−^ levels increase as a result of tellurite exposure suggesting that the stress conditions generated by K_2_TeO_3_ in *E. coli* may be associated, at least in part, with intracellular generation of superoxide.

### Tellurite induces *soxS* mRNA synthesis in *E. coli*

**Figure 5:**
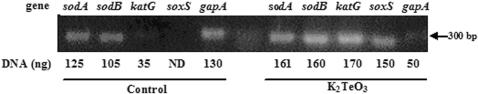
Tellurite induces *katG* and *soxS* mRNA synthesis in *E. coli.* DNA fragments (300 bp) from *E. coli sodA, sodB, katG, soxS* and *gapA* genes were amplified by RT-PCR and fractionated by electrophoresis on agarose gels (1.5%). Total RNA from cells grown with (K_2_TeO_3_) or without (control) 0.5 µg/ml potassium tellurite was used as template for the RT-PCR. The estimated DNA (ng) content determined for each band is shown (see Material and Methods for details).

To determine whether ROS-response related genes are induced by tellurite exposure, *E. coli sodA, sodB, soxS* and *katG* mRNAs were quantitated by PCR using total RNA of tellurite-treated or untreated cells. Fig. 5 shows that *E. coli sodA, sodB* and *katG* genes exhibited a rather basal expression in untreated cells. Interestingly, no *soxS* was detected under the same experimental conditions. After 10 min of tellurite exposure a marginal increase was observed for *sodA* and *sodB* mRNAs. Under the same conditions, a strong transcriptional activation was seen for *katG* and particularly for *soxS.* Only the housekeeping *gapA* gene, encoding glyceraldehyde-3-phosphate dehydrogenase, showed a transcription decrease in tellurite-exposed cells (Fig. 5). These results suggest that tellurite causes induction of genes known to be responsive to increases in ROS levels. Presence of *soxS* mRNA in tellurite-treated cells is also indicative that superoxide radical is being generated.

### Absence of *E. coli* superoxide-responsive genes causes tellurite hypersensitivity

Deletion of genes that are induced upon K_2_TeO_3_ exposure results in an increase of sensitivity to this toxic salt. Interestingly, *ibpA*-, *sodAsodB*- and *soxS*-deficient strains are the most affected, a result that agrees with observations described above. *katG*, which is highly induced in the presence of tellurite (Fig. 5) does not seem to be involved in tellurite tolerance (Table 2).

**Table 2:** Minimal inhibitory concentrations (MIC) of K_2_TeO_3 _for *E. coli* BW25113 strains deficient in ROS-responsive genes.

	Malate dehydrogenase µg/min/mg prot	Aconitase µmol/min/mg prot
Control	0.57±0.01	0.380±0.05
K_2_TeO_3_	0.59±0.04	0.085±0.02

Numbers are the mean of 4 independent determinations.

### *In vitro* superoxide generation

Results described above suggest that inside the cell K_2_TeO_3_ behaves as a ROS generator with superoxide radical being the most likely species generated. To test this assumption an *in vitro* tellurite-reduction assay was carried out (see Materials and Methods for details). The rationale for these experiments is based on previous observations indicating that superoxide radical is formed during intracellular selenite reduction [22], [23]. Because Se and Te share several chemical properties, we reasoned that tellurite reduction may also be associated with superoxide formation.

**Figure 6:**
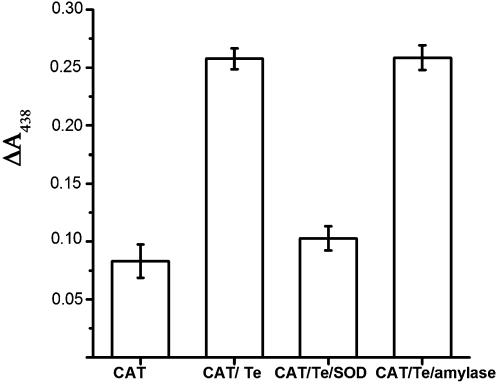
*In vitro* tellurite reduction generates superoxide in *E. coli.* Superoxide generation was evaluated using an *in vitro* tellurite reduction assay previously developed in our laboratory [34]. The system makes use of the O_2_^−^ specific reactive compound WST-1. WST-1 reduction was determined in the presence of catalase and NADPH (Cat); catalase, tellurite and NADPH (Cat/Te); catalase, tellurite, NADPH and purified superoxide dismutase (Cat/Te/SOD); catalase, tellurite, NADPH and β-amylase (Cat/Te/amylase).

As expected, *in vitro* K_2_TeO_3 _reduction resulted in an increase in WST-formazan absorption (Fig. 6); this increase was proportional to superoxide concentration (not shown). That superoxide was generated during tellurite reduction was demonstrated by performing the assay in the presence of SOD. This enzyme totally inhibited the increase of OD_438_ which did not occur when β-amylase was used. This observation confirms that superoxide radical is formed during tellurite reduction *in vitro*.

## Discussion

Results described in this work represent a step ahead in the understanding of the mechanism underlying potassium tellurite toxicity. We have previously shown that *E. coli* cells overexpressing *G. stearothermophilus* V cysteine metabolism-related genes develop a significant increase (∼25 fold) in tellurite resistance [9], [16], [24]. Expression of *G. stearothermophilus* V *cysK* and *iscS* genes prevented the typical decrease in intracellular RSH content caused by tellurite ([16] and unpublished results). Our interpretation was that the cysteine synthase-encoding *cysK* gene indirectly participates in the generation/regeneration of GSH required to maintain the reductive cytoplasmic environment altered by tellurite.

The IscS desulfurase is probably involved in recovery of tellurite-damaged [Fe-S] clusters that are part of the active site of some ROS-sensitive enzymes [9], [10]. Superoxide radical, the ROS that is most probably involved in the oxidative toxic effect of tellurite, would be generated during tellurite reduction in a process similar to that previously described for selenium oxyanions [22], [23]. ROS generation by tellurite was suggested by experiments using *sodA sodB* tellurite-hypersensitive *E. coli* mutants and by demonstration that the minimal inhibitory concentration of potassium tellurite increased under anaerobic conditions [9], [10]. We hypothesized that tellurite reduction by thiols or cellular reductases generates metallic tellurium (Te^o^) and involves ROS generation as well.

To further study tellurite-mediated toxicity we analyzed activation of stress promoters *ibpA, sulA*, P3*rpoH* and *cspA* in response to K_2_TeO_3_. Significant tellurite-mediated transcription activation was only observed for the *ibpA* promoter. The *ibp* operon is a member of the *E. coli* σ^32^ regulon that encodes the small heat shock proteins IbpA and IbpB. This operon undergoes a high level of transcriptional induction in response to a temperature up shift and participates in events associated with oxidative stress. IbpA over expression generates an important increase in resistance to superoxide anions but does not have any effect in resistance of *E. coli* to hydrogen peroxide [12], [13]. Results obtained with an *E. coli ibpA* mutant (BW25113 *kan:: ibpA*) showed a ∼32 fold increase in tellurite sensitivity as compared to the wild type strain suggesting that IbpA plays a role in K_2_TeO_3_ resistance (Table 2).

Our results showed a 2-fold increase in the *cspA* promoter transcription in response to tellurite. CspA mRNA is highly transcribed in response to cytoplasmic protein stress [14], [15]. This tellurite-mediated activation is in line with the idea that TeO_3_^2−^ has an oxidative effect on cytoplasmatic proteins by replacing Se or reacting with catalytic cysteine residues in key cellular proteins.

The P3*rpoH* promoter is induced by periplasmic protein misfolding and is transcribed by the Eσ^E^ holoenzyme [25]. We did not observe tellurite-induced activation of the P3*rpoH* promoter suggesting that tellurite-mediated stress mainly affects cytoplasmic proteins. It is well known that K_2_TeO_3_ affects proteins and cellular targets by direct interaction and/or its capability to oxidize cellular thiols [7], [16]. We hypothesized that ROS produced during Te^o^ generation could account for part of tellurite toxicity. Te^+4^ reduction takes place in the cytoplasm and it is probably mediated by thiols and/or by cellular reductases [4], [5], [26], [27]. Results of experiments with the P3*rpoH* promoter are consistent with previous findings reporting that a *Rhodobacter capsulatus* mutant in the periplasmic antioxidant gene s*enC* does not exhibit sensitivity to K_2_TeO_3_, which has been interpreted as evidence that superoxide radical is not generated in the periplasm of this rod [11].

The *sulA* gene is activated as part of the cellular SOS response induced by compounds that generate DNA damage or affect DNA replication [28]. We showed that the *sulA* gene promoter was not activated in response to tellurite suggesting that the toxic effect of tellurite does not involve DNA damage or activation of the SOS response. These results lead to the speculation that H_2_O_2_ or hydroxyl radical, that are the ROS directly involved in DNA damage [29], are not generated during tellurite reduction.

To discard a Fenton-like reaction as the one described for chromate [30] we performed *in vitro* incubations of plasmid DNA with K_2_TeO_3_ or with K_2_TeO_3_ in the presence of H_2_O_2_. No DNA damage was evidenced as judged by the lack of shift mobility on agarose gels. A similar result was obtained under tellurite reduction conditions confirming that tellurite toxicity does not seem to involve DNA damage (data not shown).

To further assess that the cellular oxidative stress generated by tellurite lies on the intracellular generation of oxygen reactive species a direct measure of cytoplasmic ROS using the intracellular, specific, probe H_2_DCFDA in *E. coli* cells treated with different sub lethal K_2_TeO_3_ concentrations was made. Tellurite treatment increased cytoplasmic ROS in a fashion that was proportional to K_2_TeO_3_ concentration (Fig. 2). These results suggested that in *E. coli* the tellurite-mediated stress can be associated, at least in part, with an increase in the cytoplasmic ROS concentration.

Protein carbonylation occurs in a number of physiological and pathological processes and it has been suggested that protein carbonylation can be used to determine the ratio between oxidative stress damage and the power of protective systems to avoid it [31]. Tellurite generates an important increase of protein carbonylation (Fig. 3A) confirming that part of intracellular toxicity involves protein damage associated to ROS generation. Oxidized proteins from cellular membranes did not show significant differences between treated and untreated cells (data not shown) suggesting that ROS generated by tellurite reduction can be associated with cytoplasmic and membrane proteins and not preferentially with membrane reductases as suggested previously [5]. Interestingly, tellurite-treated cells exhibited a higher protein carbonylation than peroxide-treated cells (Fig. 3A). These results may be related to a direct effect of tellurite on the intracellular GSH pool, which then could alter the cell's non-enzymatic antioxidant response. This would hamper the cellular defense against tellurite-induced protein oxidation through the generation of ROS or reactive aldehydes. Carbonyl generation has been associated with aldehyde-mediated protein oxidation [31]. Aldehydes are generated during lipid peroxidation, a poorly understood process in prokaryotes that may involve interactions with ROS and biological membranes.

The increase in TBARs content has been widely used to assess oxidative stress damage to lipids in many microorganisms including *E. coli* [18], [19], [31]. Fig. 3B shows that K_2_TeO_3_-treated *E. coli* cells exhibit an increase in lipid peroxidation products (determined as TBARs) suggesting that these tellurite-generated compounds are probably a consequence of ROS generation during the reduction of the tellurium oxyanion. TBARs levels were lower in tellurite-treated cells as compared to cells exposed to hydrogen peroxide (Fig. 3A and 3B). This observation may be related to the multifactor toxic effect of K_2_TeO_3_ that would affect different cellular processes through the generation of secondary toxic molecules such as O_2_^−^, aldehydes and peroxides. Preliminary results from our laboratory indicate that a putative *E. coli* aldehyde reductase can be associated with a protection against tellurite-generated lipid peroxidation products (unpublished results).

An alternative approach to monitor the generation of ROS upon tellurite exposure is to estimate induction of some specific ROS-sensitive or ROS-responsive enzymatic activities. The activity of aconitase, a superoxide sensitive enzyme, was evaluated in *E. coli* cells treated with potassium tellurite. Aconitases possess [4Fe-4S] clusters highly sensitive to superoxide and represent suitable markers to estimate ROS sensitivity. Cells exposed to tellurite exhibited an important decrease in aconitase activity (Table 2). Fumarase A, another ROS-sensitive enzyme, also showed a decreased activity under tellurite stress conditions (unpublished observations). These results confirm that tellurite triggers an enzymatic ROS phenotype, presumably via superoxide generation. The activity level of the ROS-resistant metabolic enzyme malate dehydrogenase was determined as control. No differences in malate dehydrogenase activity of *E. coli* BW25113 cells treated and untreated with tellurite was detected (Table 1). These results agree with previous observations indicating that tellurite also causes a rapid reduction of ATP levels that does not involve a decrease in the glycolytic pathway or depletion of glucose in *E. coli* [32]. This effect is most likely due to a shift to anaerobic metabolism similar to that described for other toxic metals [33].

Activation of oxidative stress enzymatic responsive systems estimated by measuring catalase (CAT) and superoxide dismutase (SOD) activities upon K_2_TeO_3_ exposure was also analyzed (Fig. 4A and 4B, respectively). Catalase HPI, encoded by the *katG* gene, is highly induced by an increase in peroxide concentration. Unexpectedly, an increase in CAT activity was observed during the first 10 min of tellurite exposure (Fig. 4A). This result may be a consequence of the hydrogen peroxide produced by superoxide dismutation because an increase in CAT activity was associated with the highest increase of SOD activity (15 min) along the K_2_TeO_3_ treatment (Fig. 4B). The fact that CAT activity does not increase at later times suggests that hydrogen peroxide is not the ROS generated in *E. coli* tellurite-stressed cells (Fig. 4A). *In situ* catalase activity determinations on native gels showed no correlation between tellurite concentration and catalase activity over 30 min incubation (data not shown). These results also favor the idea that tellurite does not produce peroxide inside *E. coli* as does the hydrogen peroxide-hypersensitive *katG* strain which showed a negligible increase in sensitivity to K_2_TeO_3_ (Table 2). *E. coli* cells over expressing a *Staphylococcus epidermidis* catalase gene showed a slight increase in tellurite resistance suggesting that the role of H_2_O_2_ in tellurite resistance, if any, is not an important feature of K_2_TeO_3_ toxicity [34].

It has been shown that SOD activity slightly increases when wt *E. coli* cells are about to enter the second half of the logarithmic growth phase [35]. We observed similar results in *E. coli* BW25113 cells that were not treated with tellurite. SOD activity was consistently induced throughout the course of the experiment confirming that superoxide dismutases play a role in the cellular response of *E. coli* to potassium tellurite (Fig. 4B).

The slight increment of *sodA* and *sodB* mRNA synthesis observed after a brief exposure to K_2_TeO_3_ also suggest that superoxide is produced in response to tellurite (Fig. 5). Induction of *katG* during early stages of tellurite treatment reflects an increase of peroxide concentration that is probably generated by superoxide dismutation as suggested above. High levels of *soxS* mRNA were also detected under tellurite exposure confirming that tellurite-generated O_2_^−^ activates the most specific and important superoxide response system in *E. coli*.

An *E. coli soxS* strain (BW25113 *kan::soxS*) showed a tellurite-sensitive phenotype (Table 2), an observation which is consistent with a role of O_2_^−^-protection systems in response to tellurite exposure. The lower sensitivity to tellurite observed by this mutant when compared with the *ibpA* and *sodAsodB* strains is presumably due to the fact that the several genes governed by the *soxRS* regulon are also regulated by other global networks [18], [36].

An *in vitro* tellurite reduction assay was used to demonstrate that reduction of Te^+4^ to Te^0^ indeed generates O_2_^−^. Production of O_2_^−^ was determined by the use of WST-1, a molecule known to be highly specific and sensitive to reduction by superoxide radicals. Enzymatic reduction of tellurite *in vitro* produced a significant increase in light absorption at 438 nm that is indicative of O_2_^−^ generation (Fig. 6). The superoxide concentration was proportional to tellurite concentration confirming that K_2_TeO_3_ reduction involves O_2_^−^ generation (data not shown). The *in vitro* reduction assay performed in the presence of SOD confirmed that superoxide is one of the products of tellurite reduction.

Altogether, our results strongly indicate that the dramatic toxic effect of potassium tellurite in *E. coli* can be associated with the chemical activity of the tellurium oxyanion at various levels of bacterial metabolic pathways including, among others, inactivation of [Fe-S] center-containing dehydratases, cytoplasmic thiol oxidation, enzyme and protein carbonylation and membrane peroxidation.

Before the submission of this manuscript Tremaroli et al. [37] inferred that superoxide radicals are generated as a result of the increased superoxide dismutase activity of *Pseudomonas pseudoalcaligenes* cells in response to tellurite exposure.

## Materials and Methods

### Bacterial strains and growth conditions

*E. coli* BW25113 [38] was the parental strain used in all experiments. *E. coli* mutants deficient in the *katG, ibpA* and *soxS* genes were provided by the NARA Institute of Science and Technology, Japan [39]. Cells were routinely grown in LB medium [40] at 37°C with shaking. Antibiotics were added as required. Kan^R^ insertions into *E. coli* BW25113 chromosomal ROS resistance genes were constructed by the method of Datsenko and Wanner [38].

### Enzyme activity assays

Cells were disrupted by sonication on ice-cooled water and extracts cleared by centrifugation. Aliquots of cell-free extracts were assayed for aconitase [20], malate dehydrogenase [21], catalase and superoxide dismutase [41], [42]. Protein concentration was determined by the Bradford method.

### Stress-promoter activation assays

Promoter regions of the *ibpA, cspA, rpoH* and *sulA* genes have been routinely used as molecular tools to detect and characterize antibacterial agents that induce stress response in *E. coli* due to their strength and specificity [43], [44].

*ibpA* gene, encoding for the small bacterial heat shock protein IbpA, undergoes high level of induction following a temperature up shift and has been associated with oxidative stress response in *E. coli* [12], [13]. CspA is the major *E. coli* cold shock protein [14]. *E. coli* cells dedicate more than 10% of their synthetic capability to produce CspA shortly after transfer to 10°C [15].

While the P3*rpoH* promoter is activated by unfolded proteins generated during periplasmic stress [25], the *sulA* gene is activated during the course of the SOS response by compounds that damage DNA or affect its replication [28].

*E. coli* strains used in the stress-promoter activation assays are ADA100 [AB734 λΦ(*ibp*::*lacZ*)], ADA310 [AB734λΦ (*cspA*::*lacZ*)], ADA410 [AB734 λΦ(p3*RpoH*::*lacZ*)] and ADA510 [AB734 λΦ(*sulA*::*lacZ*)] and were kindly provided by Dr. Francois Baneyx [43], [44].

Flasks (500 ml) containing 100 ml of LB medium were inoculated with 2 ml of overnight cultures and grown at 37°C under aerobic conditions until the optical density at 600 nm (OD_600_) was 0.4. Aliquots of 25 ml were then transferred to preheated 125 ml flasks and the cultures incubated for 3 h in the presence of 0.5 µg/ml K_2_TeO_3_. Control cultures contained an equal volume of H_2_O. The fold induction was calculated by comparing β-galactosidase activity after 3 h of tellurite treatment divided by the activity at time 0, as described by the group of Baneyx [43], [44]. All experiments were carried out in triplicate or higher.

### β-galactosidase assay

Following the incubation with tellurite, samples (2 ml) were withdrawn at defined time intervals and OD_600_ determined. Cells were sedimented by centrifugation at 6,500× *g* for 8 min, suspended in an equal volume of 50 mM monobasic potassium phosphate (pH 6.5), and disrupted by sonication. After centrifugation at 10,000× *g* for 10 min to discard cell debris, aliquots of the cleared lysate were assayed for *β*-galactosidase activity (triplicate) using the chromogenic substrate *O*-nitrophenyl-*β*-D-galactopyranoside [45].

### Tellurite sensitivity assay

Cells were grown overnight and diluted one hundred-fold with fresh LB medium. Ten µl of these dilutions were transferred into tubes containing 1 ml of LB added with specific amounts of antibiotics and potassium tellurite. Cells were incubated at 37°C for 48 h with shaking and the OD_600_ monitored to determine the minimal inhibitory concentration (MIC).

### Determination of intracellular oxidation levels

The oxidant-sensitive probe H_2_DCFDA [17] was used to determine the intracellular levels of ROS in cells treated with 3 different tellurite concentrations (0.2, 0.5, and 1 µg/ml). Cells were grown aerobically in tellurite-amended LB medium until OD_600_ 0.5, washed with 10 mM potassium phosphate buffer (pH 7.0), and incubated for 30 min in the same buffer containing 10 µM H_2_DCFDA dissolved in dimethyl sulfoxide. After washing, the cells were suspended in the same buffer and disrupted by sonication. Cell extracts (100 µl) were mixed with 1 ml of phosphate buffer (pH 7.0) and the fluorescence intensity was measured at 2 min intervals over a 30 min period using an Applied Biosystems Citofluor 4000 Fluorescence multi-well plate reader (excitation, 490 nm; emission, 519 nm). The inset of Fig. 2 represents the emission obtained for all the treatments at a fixed time (28 min). Emission values were normalized by protein concentration.

### Determination of protein carbonyl content

The carbonyl content in cellular proteins was determined as described by Semchyshyn et al. [18]. Crude extracts were prepared from *E. coli* BW25113 cells treated or untreated with K_2_TeO_3_ (0.5 µg/ml) or H_2_O_2_ (100 µM) for 30 min. Extracts were treated with streptomycin sulfate (2%) and incubated on ice for 15 min. Precipitated nucleic acids were discarded by centrifugation at 14,000× *g* for 5 min. After adding four volumes of 10 mM dinitrophenylhydrazine (DNPH, prepared in 2 M HCl) to 100 µl of the nucleic acid-free supernatant, the mixture was incubated for 1 h at room temperature with vortexing every 10–15 min. Proteins were precipitated by adding 500 µl of 20% trichloroacetic acid (TCA) and then sedimented by centrifugation at 14,000× *g* for 5 min. The pellet was washed at least three times with an ethanol:ethylacetate mixture (1∶1) to remove any unreacted DNPH and redissolved at 37°C with 450 µl guanidine HCl/dithiothreitol. Carbonyl content was determined spectrophotometrically at 370 nm using a molar absorption coefficient of 22,000 M^−1^ cm^−1^ [18].

### Determination of thiobarbituric acid-reactive substances (TBARs)

TBARs in cell extracts were determined as described by Rice-Evans et al. [19]. Briefly, 1-ml of cell suspensions were precipitated with 1.0 ml of 20% TCA (w/v) and centrifuged at 10,000× *g* for 5 min. Supernatants were mixed with 2.0 ml of a saturated solution containing thiobarbituric acid in 0.1 M HCl and 10 mM butylated hydroxytoluene. Samples were then heated for 60 min in a water bath kept at 100°C. Aliquots of 1.5 ml were then removed, chilled, mixed with 1.5 ml of butanol and centrifuged at 4,000× g for 10 min. The organic fraction was recovered and the OD_535_ was measured spectrophotometrically. TBARs content was determined using a molar extinction coefficient of 156 mM^−1^ cm^−1^ [18], [19].

### RNA purification and RT-PCR experiments

*E. coli* BW25113 cells were inoculated in two 200 ml flasks at 1∶100 dilution and incubated at 200 rpm at 37°C until the OD_600_ was 0.6. At that moment one culture was amended with K_2_TeO_3_ solution (0.5 µg/ml final concentration) and incubated for 10 min, sedimented at 13,000× *g* by 3 min and total RNA was isolated using the QIAGEN RNeasy purification kit (Promega) following the vendor recommendations. The ratio OD_260/280_ was determined for the purified RNA using an Agilent 8453 UV-visible spectrophotometer. Independent RT-PCR experiments were performed using 2 µg of total RNA as template. The set of primers used to amplify fragments of approximately 300 bp of *E. coli katG, sodA, sodB* and *soxS* genes were skatG3 5′-GCTCGCCCAACCTAAACCTTGTTC-3′, skatG5 5′-GAAAAAGCCTGGCTGACTCACCGT-3′, ssodA3 5′-TCGATAGCCGCTTTCAGGTCACCC-3′, ssodA5 5′-CCTGCCATCCCTGCCGTATGCTTA-3′, ssodB3 5′-TAAACTGCGCTTTGAAATCGGCAA-3′, ssodB5 5′-AAGATGCTCTGGCACCGCACATTT-3′, ssoxS3 5′-CGAGCATATTGACCAGCCGCTTAA-3′, and ssoxS5 5′-TTACAGGCGGTGGCGATAATCGCT-3′.

RT-PCR conditions included a 30 min incubation at 25°C with RNA-free DNAse I (Promega) followed by a DNAse-inactivating step of 10 min at 65°C. Synthesis of cDNA was allowed to proceed for 2 h at 42°C using a commercial enzyme and RT-PCR kit (QIAGEN). PCR conditions included an initial denaturation at 95°C for 5 min followed by 20 amplification cycles (95°C for 30 s, 45°C for 30 s and 72°C for 1 min). A final incubation of 72°C for 10 min was added to ensure fully extension of the amplified fragments. PCR products were fractionated on agarose gels (1.5%) and the DNA content was estimated using the Gel Pro 4.0 program (Media Cybernetics).

### Determination of *in vitro* K_2_TeO_3_ reduction reaction products

An *in vitro* K_2_TeO_3_ enzymatic reduction assay was developed to demonstrate the generation of superoxide along the course of tellurite reduction. Superoxide radical specifically reduces 2-(4-Iodophenyl)-3-(4-nitrophenyl)-5-(2,4-disulfophenyl)-2H-tetrazolium (WST-1, Cell Technology Lab) to the soluble form WST-formazan that can be easily detected at 438 nm. We have previously established that tellurite can be reduced by different enzymes including catalase [4], [26], [34]. Reduction mix contained 200 µg/ml of purified bovine liver catalase, 1 mM NADPH, 1 mM potassium tellurite and 50 mM Tris-HCl buffer pH 7.0. Generation of WST-formazan was monitored at 438 nm. Assays in which catalase was replaced by either superoxide dismutase or β-amilase were included as negative controls.
